# Emergence of Bursting Activity in Connected Neuronal Sub-Populations

**DOI:** 10.1371/journal.pone.0107400

**Published:** 2014-09-24

**Authors:** Marta Bisio, Alessandro Bosca, Valentina Pasquale, Luca Berdondini, Michela Chiappalone

**Affiliations:** Department of Neuroscience and Brain Technologies, Istituto Italiano di Tecnologia, Genova, Italy; University of Sheffield, United Kingdom

## Abstract

Uniform and modular primary hippocampal cultures from embryonic rats were grown on commercially available micro-electrode arrays to investigate network activity with respect to development and integration of different neuronal populations. Modular networks consisting of two confined active and inter-connected sub-populations of neurons were realized by means of bi-compartmental polydimethylsiloxane structures. Spontaneous activity in both uniform and modular cultures was periodically monitored, from three up to eight weeks after plating. Compared to uniform cultures and despite lower cellular density, modular networks interestingly showed higher firing rates at earlier developmental stages, and network-wide firing and bursting statistics were less variable over time. Although globally less correlated than uniform cultures, modular networks exhibited also higher intra-cluster than inter-cluster correlations, thus demonstrating that segregation and integration of activity coexisted in this simple yet powerful *in vitro* model. Finally, the peculiar synchronized bursting activity shown by confined modular networks preferentially propagated within one of the two compartments (‘dominant’), even in cases of perfect balance of firing rate between the two sub-populations. This dominance was generally maintained during the entire monitored developmental frame, thus suggesting that the implementation of this hierarchy arose from early network development.

## Introduction

A cell assembly is defined as a set of anatomically dispersed and functionally connected neurons [1]. According to Hebb's view [2], cell assemblies can be regarded as the functional unit of the brain and are characterized by coordinated activity and interaction with other groups of cells, thus supporting the hypothesis of intrinsic modularity of the brain architecture [3]. Evidence from *in vivo* studies suggests that activity of these modules plays a determinant role in any human thought or action [4], [5]. Although very informative, *in vivo* experiments do not allow controlled manipulation of the spatio-temporal dynamics of neuronal networks, while *in vitro* systems can be more easily accessed, monitored, manipulated and modeled [6]. However, in order to provide a simplified but plausible representation of interacting neuronal assemblies, *in vitro* systems should be organized in connected (‘modular’) neural sub-populations.

In recent years, *in vitro* technologies supported by advanced substrate patterning methods have made it possible to induce neuronal networks to develop a range of predefined modular structures [7] and to study the functional properties of networks with imposed topologies [8]. In the last decades, many studies have been presented, using various methods including surface modification by silane chemistry [9], photolithographic techniques [10], deep-UV lithography [11], soft lithography [12] and spot-arrays of adhesion molecules [13], [14]. Other methods were more recently used to organize networks by imposing physical constraints [15], [16]. Recently, it has been demonstrated that modular networks exhibit a large repertoire of recurring activity patterns [17]. Nevertheless, to date no systematic study on the characterization of modular networks dynamics over long time scales (i.e. months) has been reported in literature.

Here, we designed and developed a bi-compartmental system to be coupled to commercial Micro Electrode Array (MEA) substrates. Hippocampal neurons were forced to grow into two spatially–confined, rectangular, interconnected compartments of limited volume (∼0.1 µl). Once the plating and feeding protocol has been optimized, we were able to obtain long-living networks (up to 60 Days In Vitro, DIVs). We then asked whether confined modular networks so obtained followed a developmental profile similar to uniform ones. We observed that, during the early development, spontaneous activity of modular cultures is higher but globally less correlated than in uniform networks. These initial differences tended to disappear at later developmental stages. Regarding the bursting dynamics, interesting insights have been observed through the analysis of Network Bursts (NBs). It has been already demonstrated that, in bi-compartmental networks, one of the two modules plays a ‘dominant’ role (i.e. one of the two sub-networks initiates substantially more synchronized events than the other [18]). Starting from those results, here we demonstrate that NBs propagate in the same preferred sub-population for the entire development even when there is a perfect balance of firing between the two compartments.

## Materials and Methods

### Ethics Statement

As experimental model for our research we used primary hippocampal cultures from embryonic rats in view of their large diffusion as animal model in neuroscience, in particular electrophysiology and Micro-Electrode Array (MEA) research. All procedures involving experimental animals were approved by the Italian Ministry of Health and Animal Care (authorization ID 023, April 15th, 2011). When performing the experiments, we minimized the number of sacrificed rats and the potential for nociceptor activation and pain-like sensations and we respected the three R (replacement, reduction and refinement) principle in accordance with the guidelines established by the European Community Council (Directive 2010/63/EU of September 22nd, 2010). Rat housing is in accordance with the in force legislation of Italy (legislation N°116 of 1992), according to which Green Line IVC Rat cages endowed with additional environmental equipment, such as the aspen brik, are used. Regarding the husbandry conditions, the light/dark cycle is 12/12 hours, the temperature between 21–23°C and the humidity around 60%.

### PDMS structures for network confinement

The polymeric structure for the physical confinement of neuronal cultures on conventional MEAs has been realized in polydimtheylsiloxane (PDMS) by soft lithography and by using a photolithographically defined EPON SU-8 master on a silicon substrate. PDMS is an elastomer widely used for biomedical applications because of its well-known properties of biocompatibility, transparency and permeability to gases [19]; these features also render it particularly suited to the culture of primary neurons [20]. In addition, it can be easily micro-structured by soft lithography, thus obtaining several low-cost replicas from a single master [21], [22]. In our case, replicas have been produced for single use, but they could in principle be sterilized and re-used if needed.

**Figure 1:**
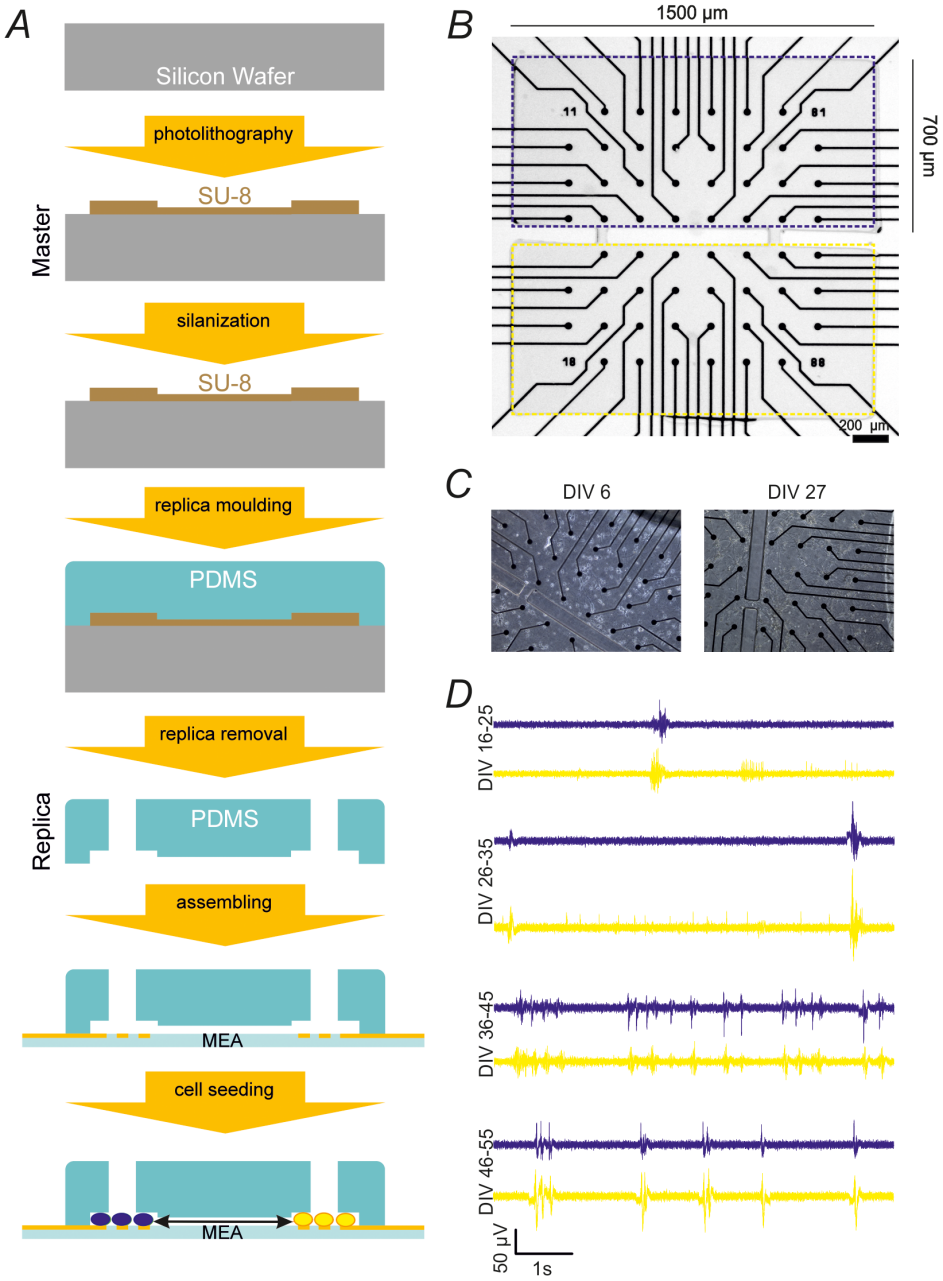
PDMS structures are used to confine neuronal cell cultures. **A**. A SU-8-on-silicon master is realized by photolithography in order to structure the polydimtheylsiloxane (PDMS) layer by replica molding. After the removal, the vias to the culturing chambers can be opened using a punch cutter and the polymeric platform can be assembled on the Micro-Electrode Array (MEA) surface using a micro-alignment system and then seeded with neural cells. **B**. Sketch of the confinement structures: two 1500×700 µm culturing chambers designed for defining two distinct neuronal populations connected through two 50 µm-wide and 100 µm-long channels; the design has been performed using professional CAD software (Silvaco Expert, Santa Clara, CA, USA). A blue dotted square highlights the top compartment, while the orange dotted square the bottom one. **C**. Optical micrograph of a modular network at two different developmental stages: 6 (left) and 27 (right) Days In Vitro - DIVs (10× magnification). **D**. Raw data recorded by two representative microelectrodes at different developmental stages. The top blue trace belongs to a representative channel in the upper compartment, while the bottom orange trace to one in the lower compartment. Duration: 6 s.

The process used to realize the PDMS platform is schematically described in Fig. 1A. Briefly, in order to create a master, a 100 µm thick layer of negative photoresist (MicroChem EPON SU-8 50) was spin-coated on a silicon wafer and structured by photolithography (MA6 aligner from Karl-Suss). The mask was realized in-house by using a direct laser writer (Heidelberg DWL 66fs), thus offering us the possibility to easily prototype different structures in short time. In order to facilitate the removal of the PDMS replicas, as well as to increase the reusability of the masters, the SU-8 structures on the silicon wafer were treated with vapor phase TCPS (trichloro(1H,1H,2H,2H-perfluorooctyl)silane). Replicas were obtained by depositing the PDMS (Dow Corning Sylgard 184) pre-polymer on the EPON SU-8 master, by curing the PDMS at 75°C for 4 hours and by carefully peeling off the PDMS layer from the mold. The so obtained PDMS structures were unmolded from the wafers and the culturing chambers were opened using a punch cutter. Finally, the PDMS structures were positioned on the MEAs (Fig. 1B) using a micro alignment system and mounted without any surface treatment in order to enable their detachment after use. In order to improve cell adhesion on the MEA surface and to avoid neuronal growth on the sidewalls of the PDMS structures, MEAs were coated with a layer of adhesion promoting poly-D-lysine (PDL) before mounting the PDMS structures. The PDMS structures were also previously sterilized in autoclave at 120°C. For cell cultures, primary hippocampal neurons were seeded inside each compartment as described in the following section.

### Experimental Protocol

#### Cleaning and sterilization of MEAs

First, MEAs had to be cleaned properly, using a soft small brush in order to remove all the residual substances from the delicate recording area. Second, they were left for one hour in distilled water added to an enzymatic active powdered detergent (Terg-A-Zyme, by Alconox, White Plains, NY 10603, USA), then they were rinsed at least twice. Once MEAs were dried, they were sterilized in the oven at 120°C for three hours. During this phase, the PDMS structures had to be prepared, following the method described in the previous paragraph.

#### Coating procedure

Once MEAs were sterilized, their surfaces had to be coated using adhesive glicoproteins in order to improve cell attachment. This procedure consisted of the following steps:

1. Deposition of a 40-µl drop of laminin solution (50 µg/ml) on the MEA recording area.
2. Incubation at 37°C for 3 hours.
3. Removal of laminin excess using 40 µl of sterile water.
4. Deposition of a 40-µl drop of poly-D-lysine (PDL) solution (100 µg/ml) on the MEA recording area.
5. Overnight incubation at 37°C.
6. Removal of PDL excess using 40 µl of sterile water.

#### Alignment of PDMS device to MEA

The alignment of the PDMS devices with the electrode array on the recording area was performed just before the plating procedure using a 5 degrees-of-freedom micro-manipulator under a stereoscope. The micro-manipulator was also provided with slide and sample holders with a vacuum attachment system, in order to lock them in the desired position during the alignment. Since this procedure was not performed in sterile conditions, once the alignment was done MEAs had to be again sterilized under UV light for 2 hours.

#### Primary neuronal cultures

Rat embryonic hippocampal neurons were obtained as previously described [23]. Briefly, embryos were recovered from CO_2_ anaesthetized pregnant rat at embryonic day 18 (Sprague–Dawley derived by Charles River in 1955, IGS). The dissociation procedure consisted of placing the hippocampus in a 5 ml Trypsin EDTA solution with the addition of 500 µl of DNAse (Sigma-Aldrich, Saint Louis, MO, USA), and leaving it for 30 minutes at 37°C. Afterwards, the solution was filled with medium (Neurobasal+B27+Pen/Strep+Glutamax, Life Technologies, Carlsbad, CA, USA) plus 10% FBS (Fetal Bovine Serum, Life Technologies, Carlsbad, CA, USA), which is a trypsin inhibitor. The solution was then centrifuged for 5 minutes at 1200 rpm, in order to completely remove the medium and add fresh one (plus 10% FBS) to the pellet. The solution was suspended again and filtered to remove clumps. Then, it was centrifuged for 7 minutes at 700 rpm to remove debris and the medium was completely removed again. Finally, fresh medium (without FBS) was added again to the pellet. Eventually, cell suspension could be diluted to reach the desired concentration. After this procedure, cells were plated on the MEAs in a short time.

#### Plating procedure

The plating procedure consisted of placing cells on the MEA recording area as the cellular suspension was ready. Since the used device had very small dimensions, it had been necessary to both compute the volume of the entire structure and to determine the optimal seeding cellular concentration that provided a high and uniform cellular density on the recording area. To do so, after several trials performed to test different seeding cellular densities, we found that the optimal concentration was about 4000 cells/µl. For seeding, a 5-µl drop of the cellular suspension at this concentration was placed in one of the two *reservoirs* and a pipette was used to allow the solution to flow from one compartment to the other, in order to guarantee the filling of the two compartments despite the high hydrophobicity of the PDMS. The approximate volume of each compartment computed accordingly to its dimensions (as specified in Fig. 1B) was equal to:
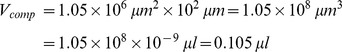



Therefore, considering that the seeding cellular concentration is 4000 cells/µl and that the compartment volume is about 0.105 µl, the approximate cell number per each compartment is around 420 cells and the estimated cellular density at the plating is 400 cells/mm^2^. Ten minutes after plating, a 100 µl drop of fresh medium was placed on the compartmentalized device surface, and the devices were left in the incubator for three hours. Successively, 900 µl of medium were added in order to provide the network with the necessary nutritive substances. For the maintenance of the cultures, a partial medium change (50%) was performed once a week.

Fig. 1C shows a network at two different stages: *i*) at an early age (left) where only the cellular bodies are visible and no connections between the two neuronal sub-populations are identified; *ii*) after 27 DIV (right), where it is possible to observe the presence of both cell bodies and neurites inside each micro-channel connecting the two neuronal populations.

For uniform networks, we followed the standard procedure already described in other papers [23], [24]. Specifically, we used a cellular solution at the concentration of 1200 cells/µl and we placed a 40-µl drop on the MEA recording area (i.e. 25 mm^2^ is the area covered by the plated drop). The final nominal density resulted to be around 1900 cells/mm^2^.

### MEA recordings

Our experimental set-up, based on the MEA 60 system, is composed of a microelectrode array, a mounting support with 60 integrated channels, a pre-and a filter amplifier (gain 1200x), a personal computer equipped with a PCI data acquisition board for real time signal monitoring and recording, an anti-vibration table and a Faraday cage. Network activity was recorded using commercial software (MCRack, Multichannel Systems, MCS, Reutlingen, Germany). To reduce thermal stress of the neurons during each experiment, MEAs were kept at 37°C by means of a controlled thermostat (MCS) and covered by a PDMS cap to avoid evaporation and to prevent changes in osmolarity [25]. Additionally, we have settled a custom incubation chamber to maintain a controlled atmosphere (i.e., gas flow of 5% CO_2_ and 95% O_2_+N_2_) during the entire recording time, as reported in previous papers [24], [26].

The spontaneous activity was monitored and recorded for 60 minutes, after a period of rest outside the incubator into the experimental set-up of 20–30 minutes, to let the culture adapt to the new environment and reach a stable level of activity [27]. Experiments were performed at various developmental stages: *i*) 16–25 DIVs; *ii*) 26–35 DIVs; *iii*) 36–45 DIVs; *iv*) 46–55 DIVs. In Fig. 1D an example of the raw traces recorded from two representative microelectrodes of a modular network during the four considered time frames is reported.

### Experimental database

Overall, we performed 58 experimental sessions equivalent to 58 hours of spontaneous electrophysiological activity recorded from 16 different cultures (6 different animals), and monitored during their development (from 16 up to 55 DIVs). Both modular and uniform networks have been monitored in order to compare their dynamics over time. A total of 8 modular and 8 homogeneous networks have been characterized from the firing and bursting point of view, each of them recorded for at least three developmental stages. For the network burst analysis, 7 modular experiments have been used. One experiment has been discarded since it did not reach the minimum threshold of 100 detected NBs, necessary for performing the statistical analysis (cf. ‘Burst, Network Burst and Burst Leaders’ paragraph).

### Data analysis and statistics

#### Spike Detection and Firing Analysis

Spontaneous spiking within the cultures is detected using the Precise Timing Spike Detection (PTSD) algorithm, accurately described and tested in a previous publication [28]. Briefly, the method uses three main parameters: *i*) a differential threshold (DT) set independently for each channel and computed as eightfold the standard deviation of the biological and thermal noise of the signal; *ii*) a peak lifetime period (PLP) set to 2 ms; *iii*) a refractory period (RP) set to 1 ms. The algorithm scans the raw data to discriminate the relative minimum or maximum points. Once a relative minimum point is found, the nearest maximum point is searched within the following PLP window (or vice versa). If the difference between the two points is larger than DT, a spike is identified and its timestamp saved.

To characterize the activity level of the analyzed neuronal networks, the mean firing rate (MFR) has been computed, which is defined as the mean number of spikes per second, computed over the total recording time (i.e. 60 min). We then evaluated the number of active electrodes inside each compartment: an even number of active electrodes indicates that the network is well balanced. We considered active electrodes as those presenting a firing rate higher than 0.01 spikes per second (sp/s). The low threshold guarantees to exclude only those electrodes that are not covered by cells or with very few spikes, keeping all the others.

#### Burst, Network Burst and Burst Leaders

A ‘population burst’ or ‘network burst’ (NB) consists of densely packed spikes occurring simultaneously at many channels, spread over the entire array. These packages generally last from hundreds of milliseconds up to seconds (burst duration) with long quiescent inter-burst periods (Inter Burst Interval - IBI). So, to investigate the bursting activity, the timing of these packages and the delay among them are fundamental variables. A custom burst detection method [29], whose input parameters were directly estimated from the inter-spike interval distribution of each channel, was used to detect bursts on single recording channels. The method exploited the logarithmic Inter Spike Interval Histogram (logISIH) to extract the parameters needed for the analysis of each recording channel. Generally, the range of ISI thresholds for our cultures is approximately 200–400 msec.

It is known from previously reported studies that a small group of electrodes leads the majority of NBs in uniform cultures coupled to MEAs [29]–[31]. Here, in confined modular cultures, we have identified the so-called Burst Leaders (BLs) as those electrodes initiating the NB, and the Major Burst Leaders (MBLs), as those channels leading at least 6% of the total number of detected NBs [30]. Additionally, one parameter has been defined and computed, namely the Network Burst Localization (NBL) index.

We then define the NBL as follows:
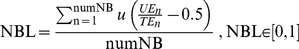
(1)where numNB is the total number of detected NB, *UE_n_* is the number of electrodes belonging to the upper compartment (taken again as an arbitrary reference) involved in the network burst *n*; *TE_n_* is the total number of electrodes involved in the *n^th^* network burst while *u()* represents the ‘step function’, which is shifted by 0.5. If 0≤NBL<0.4 NBs mostly propagate within the lower compartment, whereas if 0.6<NBL≤1 NBs mostly propagate within the upper compartment, and it is said to be a ‘NBL dominance’. On the contrary, if 0.4≤NBL≤0.6 there is no clear dominance of one of the two compartments.

#### Propagation maps

In order to analyze how NBs propagate within modular networks, the ‘propagation maps’ have been reported, considering the pattern generated by each detected MBL. Each pattern is obtained by taking into account all the other involved electrodes (‘followers’). We grouped together all bursts originated from the same leader electrode and for each follower electrode we considered the minimum propagation delay (i.e. delay of the first detected spike). In order to graphically represent the propagation pattern on the MEA layout, we reported in false colors the median of the distributions of minimum delays for each follower electrode.

#### Cross-Correlation

To study timing interactions within the monitored neuronal networks, the spike train correlation analysis was applied [32]. The cross-correlation function was built by considering the spike trains of two recording sites [33], and it is a measure of the frequency at which a spike was recorded in one recording site relative to the spike firing in another recording site, as a function of time. This function was evaluated considering all spike trains' pairs. The correlation function represents the average value of the product of two random processes (which are spike trains (), but it reduces to a simple probability C*_xy_*(*τ*) of observing a spike in one train Y at time (t+τ) given that there is a spike in a second train X at time *t* [34]; τ is called time shift or time lag. The strength of correlation between each couple of recording sites was evaluated on the basis of the peak value of the CC function, named C_peak_ [35]. Together with the C_peak_ parameter, we also evaluated the latency of the peak (i.e. L_peak_ [ms]). In order to avoid considering non-statistically significant peaks, we only selected the highest 100 C_peak_ (i.e. the strongest 100 links) for each performed experiment and their corresponding L_peak_ values lower than 50 ms. We are confident that this method underestimates the number of significant connections, but it did not include in the analysis non-statistically significant connections [35], [36].

#### Statistics

Data within the text are expressed as mean ± standard error of the mean (SEM), if not differently specified. Statistical tests were employed to assess the significant difference among different experimental conditions. The normal distribution of experimental data was assessed using the Kolmogorov-Smirnov normality test. According to the distribution of the data, we performed either parametric or non-parametric tests and p-values <0.05 were considered significant. In particular, we applied the Mann-Whitney U-test when comparing two experimental groups (e.g. modular vs uniform cultures) at each developmental stage. In order to compare cumulative distributions of the data, we employed the non-parametric two-sample Kolmogorov-Smirnov test. For multiple comparisons (e.g. same experimental group at different DIVs), we performed either the one-way ANOVA statistical test or the Kruskal Wallis Analysis of Variance on Ranks. In case of significance of the results (p<0.05), the post-hoc Dunn test was employed to assess differences. Statistical analysis was carried out by using OriginPro (OriginLab Corporation, Northampton, MA, USA) and Sigma Stat (Systat Software Inc., San Jose, CA, USA).

## Results

The experimental data presented in this report were obtained from 16 different cultures monitored from the third up to the eighth week *in vitro* once every 8–9 days. All cultures exhibited patterns of spontaneous activity, variable during their temporal maturation and depending on the plating condition (i.e. with/without PDMS structure, cf. Materials and Methods).

**Figure 2:**
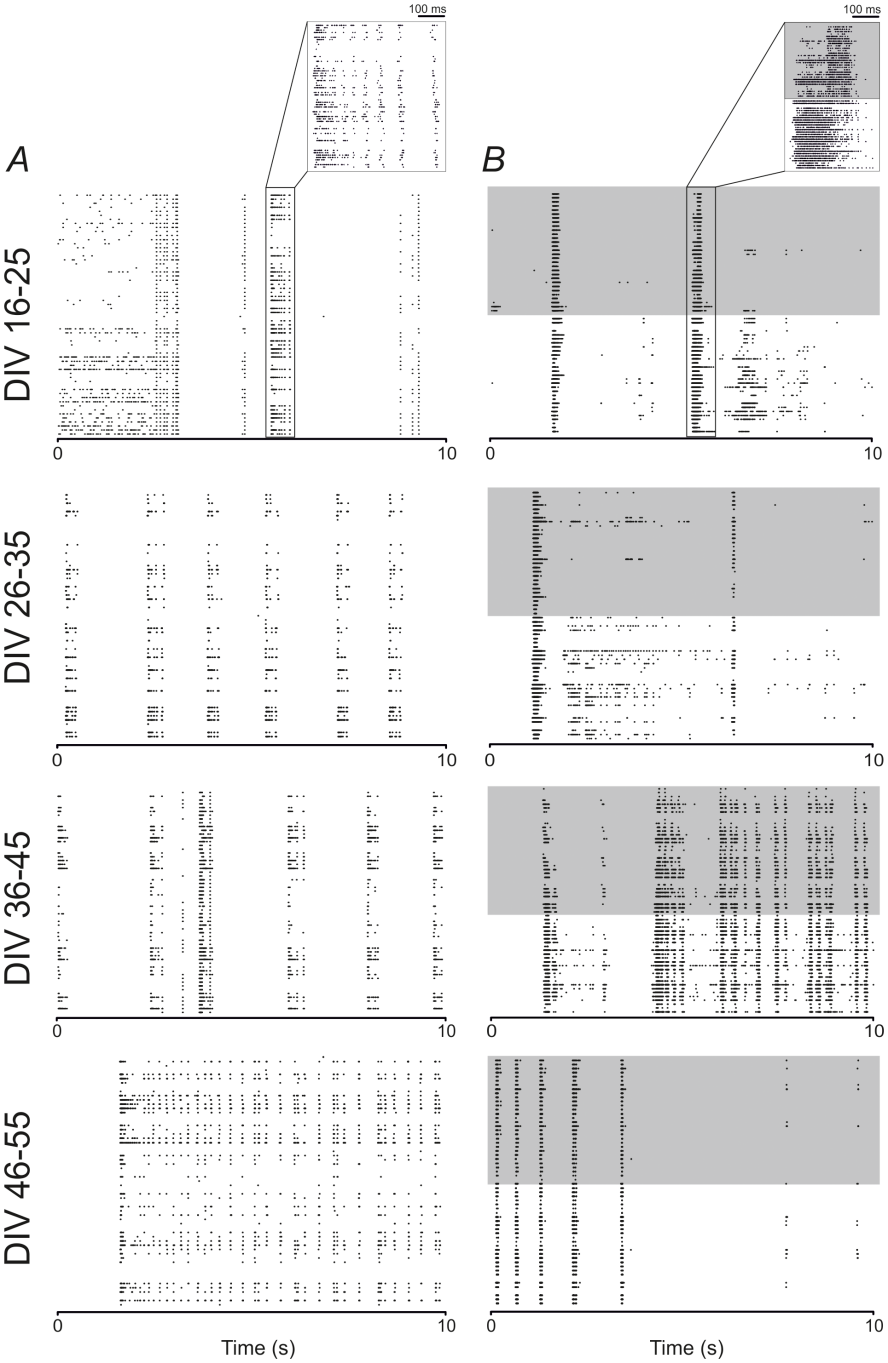
Modular and uniform neuronal networks exhibit different activity patterns during development. **A**. 10-s raster plots of spontaneous activity of a representative uniform network, recorded by 60 electrodes at four different developmental phases: 16–25 DIV, 26–35 DIV, 36–45 DIV and 46–55 DIV, respectively from top to bottom. Each black dot represents a detected spike. A zoomed network burst is reported above the raster plot at the first developmental time frame (DIV 16–25). **B**. 10-s raster plots of spontaneous activity of a representative modular network, recorded by 60 electrodes at four different developmental phases: 16–25 DIV, 26–35 DIV, 36–45 DIV and 46–55 DIV, respectively from top to bottom. Each black dot represents a detected spike, and the network confinement is highlighted by the grey shaded area, which corresponds to the top compartment. A zoomed network burst is reported above the raster plot at the first developmental time frame (DIV 16–25).

Fig. 2 shows the raster plots depicting a 10-s long spontaneous activity period recorded by two representative cultures during their *in vitro* development. A uniform (i.e., without the PDMS structure - Fig. 2A) and a modular (i.e. with the PDMS structure - Fig. 2B) network are presented at the four considered developmental stages: DIV 16–25; DIV 26–35; DIV 36–45; DIV 46–55. During the first time frame (DIV 16–25), both networks show a mix of sparse spiking and synchronized bursting. In the time course of the development (DIV 26–35, 36–45, 46–55), the behavior of the uniform network is in line with previously reported data [37], [38] and all recording channels appear strongly synchronized. In the modular network, differences between the top (grey shaded in Fig. 2B) and the bottom compartment are clearly visible since the bottom compartment is more active (i.e. it presents a higher density of random spikes), especially in the first two time frames (i.e. DIV 16–25 and 26–35). For the first DIV range also a zoom of a network burst is reported above each raster plot in order to show how the pattern propagates within each network. It is worthy of note a delay in the propagation pattern between the two compartments within modular networks. Such a delay is instead not observable in uniform cultures. As the modular network gets older, synchronization increases, and the differences between the two compartments disappear in the last monitored stage (DIV 46–55). As a general observation, the level of activity looks higher for young modular than for young uniform networks.

**Figure 3:**
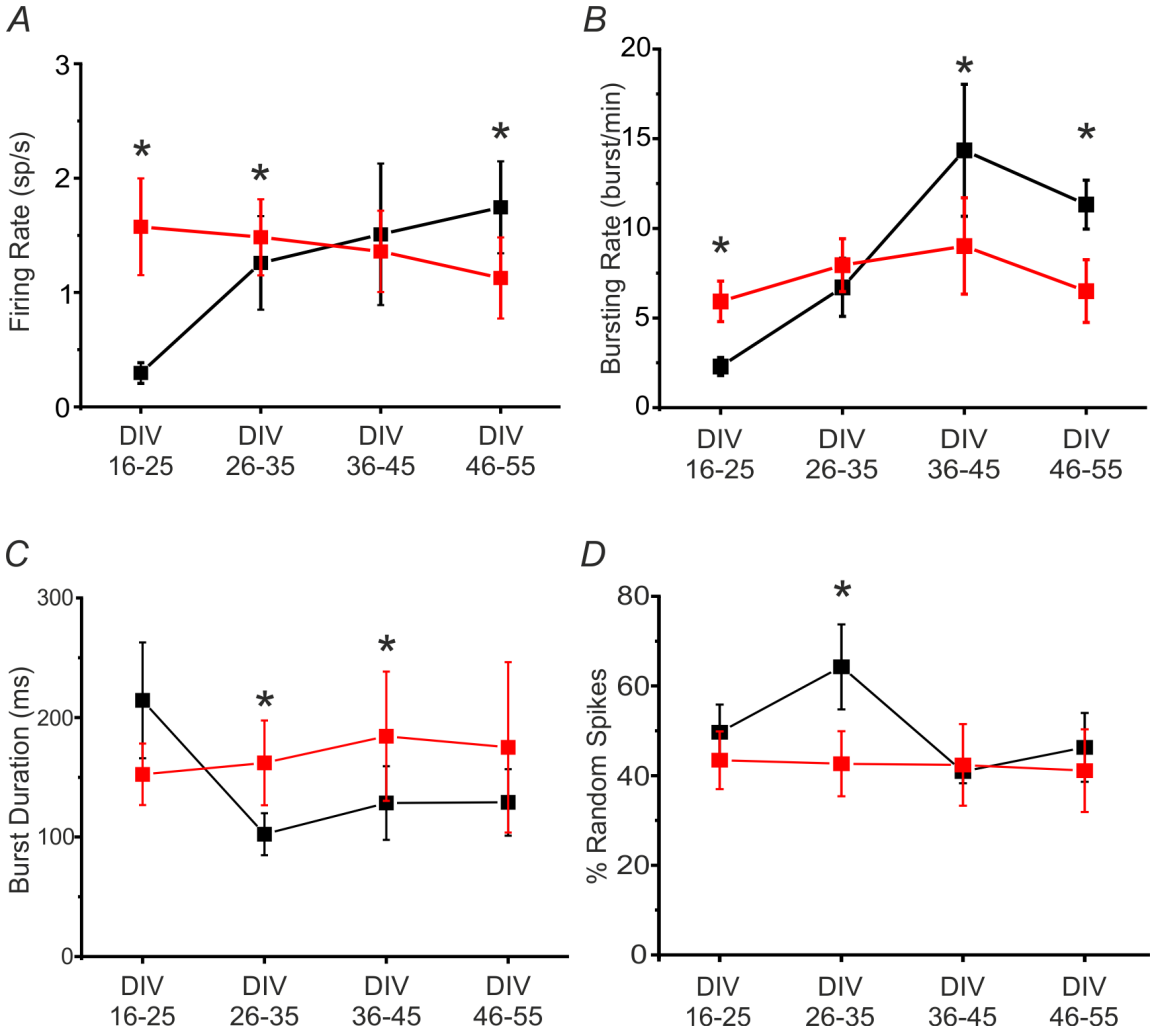
Modular and uniform networks show different developmental profiles. **A**. Firing rate (sp/s) of 8 modular (red) and 8 uniform networks (black). The parameter is statistically different between the two groups at the following DIV ranges: 16–25 DIV, 26–35 DIV and 46–55 DIV. **B**. Bursting rate (burst/min) of modular (red) and uniform networks (black). The parameter is statistically different between the two groups at the following DIV ranges: 16–25 DIV, 36–45 DIV and 46–55 DIV. **C**. Burst duration (ms) of modular (red) and uniform networks (black). The parameter is statistically different between the two groups at the following two DIV ranges: 26–35 DIV and 36–45 DIV. **D**. Percentage of random spikes of modular (red) and uniform networks (black). The parameter is statistically different between the two groups at only one DIV range (26–35 DIV). All data are presented as mean +SEM. Statistical analysis has been performed by using the Mann-Whitney U test [*p<0.05].

Typical network parameters, referred to the spiking and bursting activities exhibited by the measured cultures, have been computed for all monitored time frames. Fig. 3 compares the firing and the bursting rates of modular and uniform networks during their development. The two types of networks follow a different developmental profile for both parameters, with diverging values at the beginning and at the end of the development. In the central period of the development (DIV 36–45 for firing and DIV 26–35 for bursting), the behavior of the two network types tends to be similar (Fig. 3A and B). It is worth noting that modular networks have a higher firing/bursting rate compared to the uniform ones at the very beginning of their development (Fig. 3A), as qualitatively observed also in the raster plots of Fig. 2. Modular networks per se show a quasi-flat firing and bursting profile during the entire development, as confirmed by statistical analysis (Kruskal Wallis One Way Analysis of Variance on Ranks: p = 0.815, 3 Degrees of Freedom - DF- for the firing rate profile; p = 0.859, 3 DF for the bursting rate profile). Regarding uniform networks, the firing and bursting rate profiles change significantly during development (Kruskal Wallis One Way Analysis of Variance on Ranks: p = 0.044, 3 DF for the firing rate profile; p = 0.003, 3 DF for the bursting rate profile). In particular, for the firing rate we found a significant difference between the 16–25 DIV range vs all the others. For the bursting rate, statistical significance was found between the 16–25 DIV range and the 36–45 and 46–55 DIV only.

The variability of the parameters was monitored through the computation of the coefficients of variation (CV, defined as standard deviation of the mean divided by the mean) for both network types [39]. The CV of the firing rate (CV_FR_) for the uniform networks is 0.92±0.28 (Standard Deviation - SD), while for the modular is 0.70±0.05 (SD). The CV_FR_ is lower in case of modular networks during the entire development and presents a lower variation compared to uniform networks (except for the last developmental point, data not shown). The CV_BR_ (i.e. CV of the bursting rate) for the uniform networks is 0.53±0.19 (SD), while for the modular is 0.61±0.12 (SD), indicating a comparable level of variability between the two networks.

Fig. 3C and D show, respectively, the mean duration of bursts and the percentage of random spikes (i.e. percentage of spikes outside the bursts) for the two networks. Modular networks have a quite constant behavior for these two parameters, presenting a flat curve, as confirmed by statistical analysis (Kruskal Wallis One Way Analysis of Variance on Ranks: p = 0.987, 3 DF for burst duration profile; p = 0.960, 3 DF for the percentage of random spikes profile). Uniform cultures, at first glance, follow a different profile: the burst duration is high in the first time frame and then it decreases remaining stable for the rest of the development, but this difference is not statistically confirmed (Kruskal Wallis One Way Analysis of Variance on Ranks: p = 0.266, 3 DF). Also the percentage of random spiking tends to remain stable for the entire development (Kruskal Wallis One Way Analysis of Variance on Ranks: p = 0.307, 3 DF). The CV has been computed also for these parameters. The CV of the burst duration (CV_BD_) for the uniform networks is 0.53±0.08 (SD), while for the modular is 0.70±0.19 (SD). The CV_BD_ is lower in case of uniform networks during the entire development and presents a lower variation compared to modular networks. The CV_RS_ (i.e. CV of random spiking) for the uniform network is 0.32±0.14 (SD), while for the modular is 0.49±0.06 (SD). CV_RS_ is higher for the modular than the uniform networks, but more stable during the entire developmental frame.

**Figure 4:**
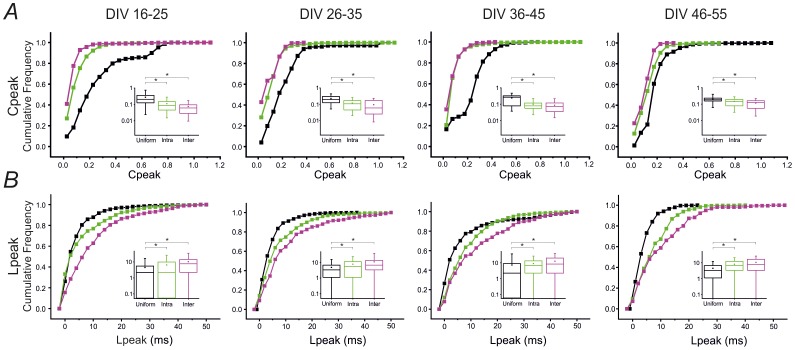
Modular cultures are globally less correlated than uniform and show higher intra vs inter correlation. **A**. Cumulative frequency graphs making a comparison, at each developmental time frame, between the following distributions of the 100 strongest C_peak_ values computed from the cross-correlograms of each pair of active electrodes: uniform (black curve), intra-compartmental (green curve) and inter-compartmental (magenta curve). Insets: box plots showing the three corresponding distributions. **B**. Cumulative frequency graphs making a comparison, at each developmental time frame, between the following distributions of the peak latency values corresponding to the pre-selected 100 strongest C_peak_ values: uniform (black curve), intra-compartmental (green curve) and inter-compartmental (magenta curve). Insets: box plots showing the three corresponding distributions. Statistical analysis has been performed on 8 uniform and 8 modular networks by using the two-sample Kolmogorov Smirnov test [*p<0.05].

We then investigated whether and how the correlation level changes during the development (cf. Fig. 4), by considering the cumulative distribution of C_peak_ and L_peak_ values computed from the cross-correlograms of each pair of active electrodes (cf. Materials and Methods). Fig. 4A compares the correlation peaks of uniform networks (black curve) with the local intra-compartmental (green curve) and inter-compartmental (magenta curve) correlation peaks of modular networks. The graph indicates that inter-compartmental correlation peaks (i.e. Inter) have lower values compared to the intra-compartmental (i.e. Intra) and the uniform ones. This difference is also noticeable when looking at the inset box plots. This means that electrodes belonging to the same compartment are more correlated, thus highlighting the confinement effect of the mask on the network dynamics. Fig. 4B, instead, shows the cumulative distributions of the L_peak_ values. Longer latencies for the inter-compartmental case are observed, thus suggesting a delay in the activity propagation between the two compartments. The insets in the panels of Fig. 4B show the box plots, which better highlight the differences between the three distributions.

A two-sample Kolmogorov Smirnov test has been used in order to statistically analyze the differences between the following distributions pairs: uniform versus intra-compartmental and intra-compartmental versus inter-compartmental for both the C_peak_ and L_peak_ distributions. So, we can assert that these distributions pairs are always statistically different for all studied developmental time frames (two-sample Kolmogorov Smirnov, p<0.05).

These results confirm the qualitative results of the raster plots. Indeed, since uniform networks are always more correlated, they show a more synchronized activity since the very beginning, while modular networks show two different levels of synchronization due to the confinement, which allows the formation of localized connected circuits mostly inside each compartment.

The previous results underline the main differences between uniform and modular networks in terms of firing and bursting dynamics during the *in vitro* development. As a second step, we were interested in better understanding how synchronized patterns of activity (i.e. NBs) are generated and propagate within modular networks during development.

**Figure 5:**
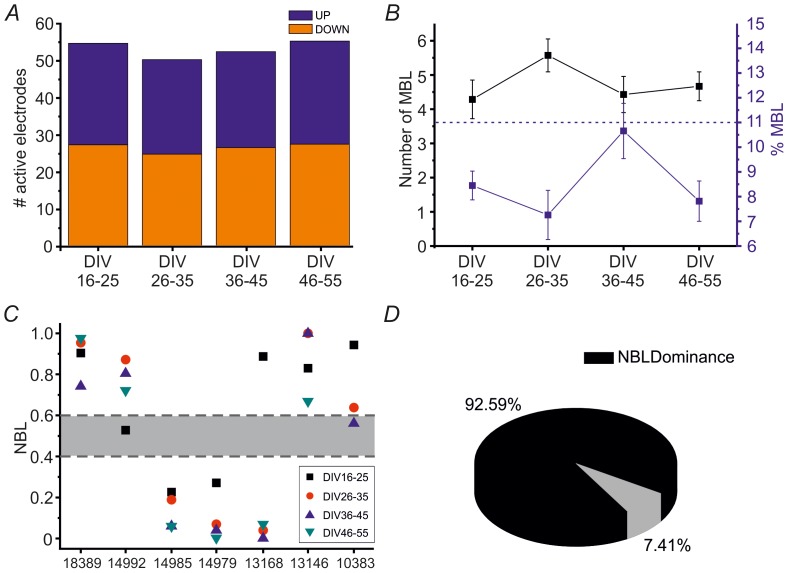
Network Bursts mostly propagate in one compartment (‘leader’) during development. **A**. Bar plot showing the mean number of active electrodes within the lower compartment (orange bars) and within the upper compartment (blue bars) at different DIV ranges (horizontal axis). Among the four groups there is no statistically significant difference (one-way ANOVA, p>0.05). **B**. Number of Major Burst Leaders (MBL, black line) and percentage of MBLs over the total number of active electrodes (blue line) as a function of time during development. The dotted blue line represents the percentage of MBLs estimated for uniform hippocampal cultures. Data are presented as mean ±SEM. (one-way ANOVA, p>0.05 for both the black and the blue profile). **C**. Network Burst Localization parameter (NBL – vertical axis, cf. Eq. 3), computed per each DIV range for each performed experiment on modular cultures (horizontal axis). The values corresponding to the different developmental stages are reported in different shapes and colors, as indicated by the legend. The grey area indicates the confidence interval (0.4 ÷ 0.6). We selected here only those modular cultures (7 out of 8) that exceed the threshold value imposed for the number of detected NBs. **D**. Pie chart reporting the percentage of recordings showing ‘NBL dominance’ (in black, cf. Material and Methods) or showing NBL values lying inside the confidence interval of 0.5±0.1 (in grey).

First, we aimed at verifying whether the network activity is equally distributed in both compartments. In Fig. 5A the orange bars indicate the mean number of active electrodes detected in the lower compartment while the blue ones the mean number of active electrodes detected in the upper compartment, both computed by taking into account all performed experiments at each developmental time frame. It is possible to observe that the two different color bars are approximately the same height at each time frame, thus indicating a comparable number of active electrodes in both compartments with very small variability during the development. Since both compartments have roughly the same number of electrodes recording firing activity, this indicates that the presence of the physical constraint due to the PDMS mask does not prevent cells from equally distributing on the entire recording area.

Before proceeding in investigating the NB dynamics, we wanted to analyze how many channels were involved in the generation of the NBs. To do so, the Major Burst Leaders (MBLs, the most frequent ignition sites of the bursting activity – cf. Materials and Methods) have been identified. Fig. 5B reports the mean number of MBLs (black line) and the percentage of MBLs over the total number of active electrodes (blue line), computed for all the considered developmental stages. The percentage of MBLs for hippocampal uniform networks is also reported on the same graph (horizontal blue line at 11%). It is possible to observe that the mean number of MBLs does not significantly change during development (one-way ANOVA, p = 0.287), and that the percentage of MBLs is upper limited by 12%. Since the number of MBLs identified per each culture is low compared to the number of its active electrodes (cf. Fig. 5B), it is possible to conclude that there is a small group of electrodes leading the entire network activity during the development also in confined modular cultures.

We then asked whether the so generated NBs preferably remained in the same compartment or propagated towards the other one. Fig. 5C shows the Network Burst Localization parameter (NBL, cf. Material and Methods, Eq.3) for the seven considered modular networks. As reported in the Methods section, the NBL indicates where the different NBs mostly propagate. The graph indicates that each culture has indeed a ‘dominant’ module that remains the same during the entire development, except for two cultures which do not present a dominant compartment, respectively at DIV 16–25 and DIV 46–55. Fig. 5D shows the percentage of experiments in which there is a clear-cut ‘NBL dominance’, with respect to the total number of performed recordings (i.e. 27, considering all the DIV frames at which each modular network has been monitored).

**Figure 6:**
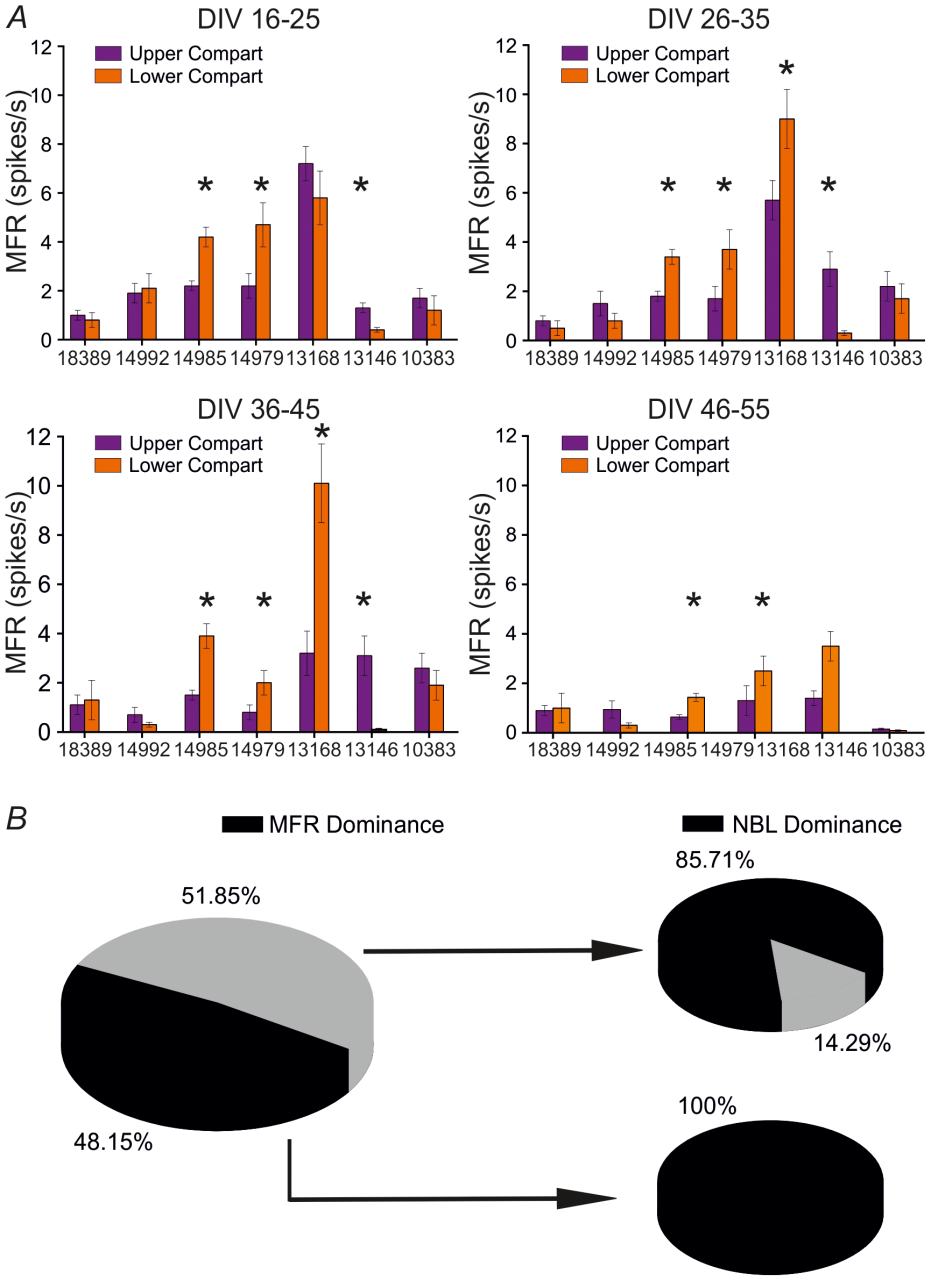
Network bursts propagation dominance is independent from the balance of firing rates between the two compartments. **A**. Average MFR computed inside each compartment (i.e. the upper one in magenta, and the lower one in orange) for all the performed experiments (horizontal axis). Each graph corresponds to each studied developmental time frame. All data are presented as mean ±SEM. Statistical analysis has been performed by using the t-test [*p<0.05]. **B**. *Left*. Percentage of times in which there is a dominant compartment (i.e. there is a statistical difference between the two compartments' level of firing) with respect to the total number of performed experiments (in black) and percentage of times in which there is not a dominant compartment with respect to the total number of performed experiments (in grey). *Right*. The upper pie chart reports the percentage of times in which there is ‘NBL dominance’ or not (cf. Material and Methods), given that there is not statistically significant difference between the two compartments' firing, whereas the lower pie chart the percentage of times in which there is ‘NBL dominance’, given that there is a dominant compartment from the firing rate point of view.

We then decided to investigate more in detail if there is a relationship between the level of firing of each compartment and the NBL dominance. Fig. 6A shows the average MFR computed inside each compartment (i.e. the upper one in magenta, the lower one in orange) per each performed experiment, for the entire monitored period. We performed a t-test in order to assess whether or not there is a statistical difference between the upper and the lower compartment's firing, for all the considered cases. Fig. 6B on the left shows the percentage of recordings in which one compartment is more active than the other (in black), and the percentage of recordings in which both compartments have the same level of firing (in grey). This panel summarizes data reported in Fig. 6A and shows that there is a roughly equal probability of finding balance or unbalance of firing rate between the two compartments. Then, for both cases we analyzed the ‘NBL dominance’. The pie charts on the right show the percentage of recordings in which there is ‘NBL dominance’ (in black) or not (in grey), given that there is (lower right pie) or not (upper right pie) statistical difference between the two compartments' firing activity. In both cases, the percentage of recordings showing ‘NBL dominance’ is markedly higher than that of recordings showing no propagation dominance. These results indicate that, considering a uniform covering of the recording area, as shown in Fig. 5A, there is a ‘preferred’ module in which NBs mostly propagate for the entire development. Interestingly, this dominance is observed also when a perfect balance of firing rate among the two compartments is found.

**Figure 7:**
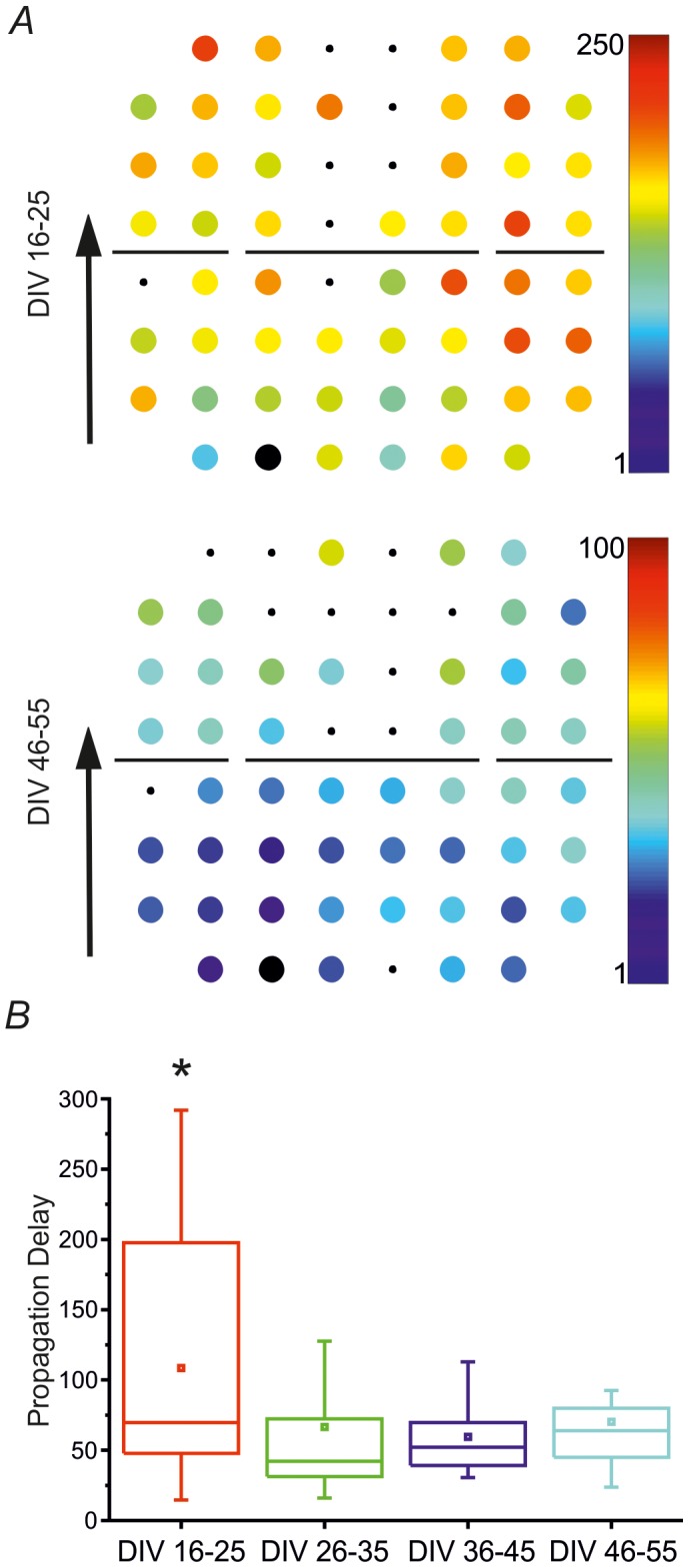
Network Bursts propagate with longer time delays at the beginning of the studied developmental stage. **A**. Propagation maps obtained from the analysis of MEA no. 14985, showing the pattern generated by MBL no. 38. *Top panel*. A representative propagation map obtained for MBL 38 at the first developmental stage (DIV 16–25): the maximum temporal delay for this pattern is 209.75 ms. *Bottom panel*. A representative propagation map obtained for MBL 38 at the last developmental stage (DIV 46–55): the maximum temporal delay for this pattern is 58.15 ms. In the two panels, NBs are both generated and propagate within the lower compartment (i.e. NBs mostly initiate from channels no. 27, 28, 37 and 38, during the entire network development). The big black dot in each map represents the electrode from which NBs generate (i.e. the MBL). **B**. Statistical distributions of maximum propagation time delays (Δt_max_) computed per each developmental stage. Modular networks present longer propagation delays at the beginning of the development, which decrease after the fourth week *in vitro*. The values reported in the graph are the following: *i*) 16–25 DIVs: Δt_max_ = 108.45±14.95 ms; *ii*) 26–35 DIVs: Δt_max_ = 66.50±8.44 ms; *iii*) 36–45 DIVs: Δt_max_ = 59.53±4.68 ms; *iv*) 46–55 DIVs: Δt_max_ = 70.14±6.81 ms. This box chart has been obtained from 7 different experiments. Statistical analysis has been performed by using the One Way ANOVA test [*p<0.05].

Fig. 7A reports the propagation maps of the NBs starting from a single MBL belonging to the lower compartment (electrode no. 38, third column and eighth row, indicated in the layout by a black dot), showing how network bursts propagate within the two compartments (cf. Materials and Methods) at an initial developmental stage (top) and at the end of the development (bottom) for MEA 14985. We can point out that, in both cases, NBs generate and mostly propagate within the lower compartment which is constantly identified as ‘leader’ throughout the development. This result is confirmed by looking at the NBL values for experiment 14985 in Fig. 5C.

Fig. 7B reports the mean value and the standard error (SEM) of the maximum time delay (Δt_max_) of each pattern per each developmental stage. They are, on average, longer at the beginning of the network development and tend to decrease in older cultures, as it emerges by looking at the temporal color scale beside each reported map. Indeed, in Fig. 7A the bottom figure (i.e. late development) presents a pattern mostly colored in blue (i.e. short delays).The post-hoc test indicated that the first experimental group is significantly different from the second, the third and the fourth, thus confirming that modular networks present longer propagation delays at the beginning of the development (third week *in vitro*), which then decrease later on.

## Discussion

In this paper we developed an effective approach and cell culture methodology to grow and structure neuronal networks with the aim of investigating whether and how modularity, which also implies confinement, influences the generation and propagation of activity during network maturation. In order to respond to our question, we first improved the MEA technology to allow long-term stable measurements of modular networks; we then compared the spontaneous dynamics of modular vs uniform cultures on a long time frame (up to eight weeks *in vitro*); finally, we focused on the emergence of collective bursting behavior in modular assemblies.

### Technological approach

From the technological point of view, we developed a micro-machined polymeric structure that can be easily mounted and removed from MEAs, thus enabling its re-use and potentially the mounting of different network structuring devices for sequential experiments. Similar approach has been already presented by previous studies [40], but in this work it was improved to allow the growth of modular networks by using the same surface functionalization as done for uniform cultures. This strategy gave us some advantages with respect to previously presented methodologies. Specifically, it provided us with a more stable and reliable long-term confinement [41] with respect to strategies based on chemical patterning [9], [10], [12]–[14], [42]. Moreover, our choice, that required a careful optimization of the coating, assembly and sterilization procedures as well as the implementation of a custom micro-alignment system, allowed the long-term survival of our cells (i.e. up to 8 weeks *in vitro*), a result never reported in other studies adopting a similar methodology. Our approach also avoided the tendency of cells to grow on the edges of the confinement structures, since the polymeric structures were not coated with adhesion molecules. Finally, the precise design and micro-fabrication of the confinement structures, differently from other works [17], [18], guaranteed the reproducibility among different samples reinforcing our statistical analysis.

### Differences between modular and uniform cultures

To compare the spontaneous dynamics of confined modular vs uniform cultures, we performed experiments on 16 cultures (8 modular and 8 uniform) monitored from 16 up to 55 DIVs, divided into four DIV ranges: *i*) 16–25 DIVs; *ii*) 26–35 DIVs; *iii*) 36–45 DIVs; *iv*) 46–55 DIVs. Here, the novelty consists in the comparison between the properties of confined modular and uniform cultures over an 8-week time frame, during which both kinds of cultures are maintained in healthy conditions. We then asked whether modularity can influence the development of the network's spontaneous activity.

When comparing the two groups (i.e. modular vs uniform), we observed differences in terms of activity parameters during the *in vitro* development. Modular networks showed higher firing and bursting rates at earlier developmental stages (16–25 DIV) (cf. Fig. 2 and Fig. 3). This can be due to several factors. First, at the beginning of *in vitro* development the restricted area might favor the formation of early synaptic connections, despite the lower cellular density of modular cultures (400 *vs* 1900 cells/mm^2^). Secondly, the reduced volumes of the two compartments might also ease the network's maturation, especially at the beginning of the development, by reducing the dispersion and enhancing the accumulation of growth factors. Finally, we must consider that modular networks, because of the presence of the PDMS mask, are prevented from extending outside the recording area, as it happens in unconstrained networks, where we have large portions that are not monitored: this strongly reduces the under-sampling phenomenon. This fact could in principle increase the differences observed at the beginning of development, when activity is definitely sparser than at later stages. Despite confined modular networks present a lower cellular density (400 *vs* 1900 cells/mm^2^) and a lower network size (approx. 420 *vs* 48,000 cells) with respect to uniform cultures, we do not think that these factors can solely account for the observed differences in the developmental profile of firing activity. In fact, previous studies analyzed changes in neuronal network dynamics as a function of network size [6], [43] and cellular density [44]. All these studies concluded that firing and bursting rate generally increase proportionally to these two culturing parameters, leading us to expect to observe lower firing and bursting rates in confined modular cultures (because of the lower size and density) than in uniform ones. However, we obtained opposite results especially at the beginning of the analyzed developmental frame. Hence, we are convinced that the reasons of these differences must be searched in the factors listed above.

Therefore, our conclusion is that the presence of the mask, which forces neurons to grow and extend in a restricted area/volume and reduces under-sampling, has the effect of accelerating the developmental processes with respect to unconstrained uniform cultures, despite the reduced cellular density and network size. Of course, in order to test this hypothesis we should better focus on the very first period of development (7–15 DIV, 2^nd^ week *in vitro*) when synaptogenesis start to take place and the electrophysiological activity can be also monitored. Additionally, it would be interesting to evaluate differences between compartmentalized and uniform networks with equal cell density (i.e. 400 cells/mm^2^). We would expect to find a similar developmental profile for the uniform ones, but with lower firing and bursting rate levels as reported in previous studies [38], [44]. A further extension of our study could be associated with the variation of the compartmental size and the number of compartments. It has been already observed for isolated clusters (i.e. ‘finite-size’ networks) that the frequency of synchronous network events increased with circuit size [6], [43]. An interesting prosecution of our work would consist of employing a higher number of interacting modules in order to analyze the onset of more complex dynamics, and the emergence of different hierarchical structures. We reserve to more deeply investigate these phenomena in a future work.

In addition, our results (cf. Fig. 3) indicate that the firing and bursting dynamics of modular networks is not affected in a significant way by the time course, differently from what reported for uniform cultures [37], [38]. After the initial divergence, modular and uniform networks display comparable values of firing/bursting rates (cf. Fig. 3A–B, DIV ranges 26–35), in accordance with results already published in the literature referring to a similar culturing condition for cortical networks [45]. At the end of the monitored period (36–45 and 46–55 DIVs), uniform networks are more active than modular, presumably due to the fact that their size is larger and the average number of synapses per neuron at steady-state could in principle be much higher than in smaller modular networks [38].

Differences in the network dynamics of the two groups are also supported by the cross correlation analysis (cf. Fig. 4). Globally, uniform networks are characterized by a higher level of cross-correlation than modular cultures. This difference is attenuated during development but also holds for older cultures (46–55 DIVs). Moreover, for modular networks, a lower inter-compartmental correlation is always observed compared to the intra one during the entire development, which suggests de-correlation of activity imposed by the physical constraint. This result confirms previous findings reported in the literature, according to which each sub-network, being part of a modular network, exhibits higher levels of internal connectivity compared to the level of connectivity between the two sub-networks [18], [46].

### Propagation of Network Bursts in modular cultures

Finally, we investigated the propagation of the peculiar synchronized bursting activity shown by modular cultures. Altogether, the results of Fig. 5 indicate that generally NBs propagate within a ‘preferred’ compartment for the entire development. Interestingly, modular networks present a ‘preferred’ or ‘dominant’ compartment even when there is a perfect balance of firing rate among the two neuronal sub-populations (Fig. 6).

In the literature, it has been already reported that in interconnected cortical cultures there is an asymmetry in the generation and propagation of activity, displaying a master-slave relationship which seems to be an innate property of these networks [18]. One of the two sub-populations (the leader or master) initiates more mutual network bursts than the other (the follower or slave). Our results confirm the dominant role of one of the two sub-networks also in hippocampal cultures and extend this concept for the entire duration of the studied culture development. These results are reliable since we demonstrated that the number of active electrodes in either compartment, involved in the spiking and bursting activity, is roughly the same and remain stable during the entire network development.

Since the same ‘leader’ compartment is identified at each studied developmental frame for almost all the experiments, it is possible to assess that a fixed hierarchy is already present in modular networks at early development (DIV 16–25) thus suggesting a previous self-organization of the network's geometry thanks to the localized area inside which neurons are forced to grow.

Furthermore, comparing the NB propagation patterns at the beginning and at the end of development, we found another correspondence between our results and the literature. In fact, it has been already demonstrated that NBs' propagation delays are longer at the beginning of development than at the end [31]. This is also observable from our results, where a considerable decrease of temporal propagation delays is evident starting from the second considered developmental frame (cf. Results and Fig. 7). This result is also confirmed by the already discussed increase of correlation between the two compartments activities along the development, thus suggesting a simultaneous decrease in the temporal propagation delays of the signal between the two neuronal sub-populations.

In conclusion, we have shown that through our technical approach we can obtain long-lasting, reproducible and stable engineered neuronal networks. Moreover, our results on the hierarchical organization of neural networks suggest that the critical period for implementing this network organization could be the early phase of network formation. Indeed, our results originally demonstrate that once this organization is set it is preserved all along the developmental frame.
